# The Retinal Vasculature and Risk of Age-Related GFR Decline — The Renal Iohexol Clearance Survey

**DOI:** 10.1016/j.ekir.2025.02.006

**Published:** 2025-02-14

**Authors:** Silje Småbrekke, Karl Marius Brobakk, Nikoline B. Rinde, Therese Von Hanno, Geir Bertelsen, Bjørn Odvar Eriksen, Toralf Melsom

**Affiliations:** 1 Metabolic and Renal Research Group, UiT The Arctic University of Norway, Tromsø, Norway; 2 Department of Clinical Medicine, UiT The Arctic University of Norway, Tromsø, Norway; 3 Section of Radiology, University Hospital of North Norway, Tromsø, Norway; 4 Section of Nephrology, University Hospital of North Norway, Tromsø, Norway; 5 Department of Ophthalmology, Nordland Hospital Trust, Bodø, Norway; 6 Department of Community Medicine, UiT The Arctic University of Norway, Tromsø, Norway; 7 Section of Ophthalmology, University Hospital of North Norway, Tromsø, Norway

**Keywords:** CRAE, CRVE, GFR decline, measured GFR, nondiabetic, retinopathy

## Abstract

**Introduction:**

Age-related decline in glomerular filtration rate (GFR) significantly contributes to chronic kidney disease (CKD). This longitudinal study in a nondiabetic population investigated whether retinal microvascular changes are associated with GFR decline.

**Methods:**

The Renal Iohexol Clearance Survey (RENIS) included 1837 participants aged 50 to 62 years without self-reported diabetes, kidney or cardiovascular disease. Baseline retinal vessel measurements and retinopathy were assessed with a Visucam PRONM retinal camera. Iohexol clearance was measured over 1 to 4 visits across an 11-year median follow-up. Linear mixed models and logistic regression were used to analyze associations between retinal vessel measurements, retinopathy, mean annual GFR decline, and accelerated GFR decline.

**Results:**

In multiple adjusted linear mixed models, wider central retinal venular equivalent (CRVE) and wider central retinal arteriolar equivalent (CRAE) were associated with a steeper mean measured GFR (mGFR) decline. For each SD increase, CRVE was associated with an mGFR decline of −0.08 ml/min/yr (95% confidence interval [CI]:−0.15 to −0.02; *P* = 0.012), and CRAE was associated with a decline of −0.09 ml/min/yr (95% CI:−0.15 to −0.02; *P* = 0.007). CRVE, but not CRAE, was associated with accelerated mGFR decline in the model adjusted for age, sex, and height [OR 1.31 (95% CI 1.07-1.61, *P* = 0.008]. No significant associations were observed between retinopathy, microaneurysms, and hemorrhages with annual or accelerated mGFR decline.

**Conclusion:**

CRVE and CRAE, but not retinopathy, retinal microaneurysms, or hemorrhages, were associated with steeper mean mGFR, suggesting that microvascular changes may be one of the underlying mechanisms for age-related GFR loss in a general nondiabetic population.

## Graphical abstract

**Figure:**
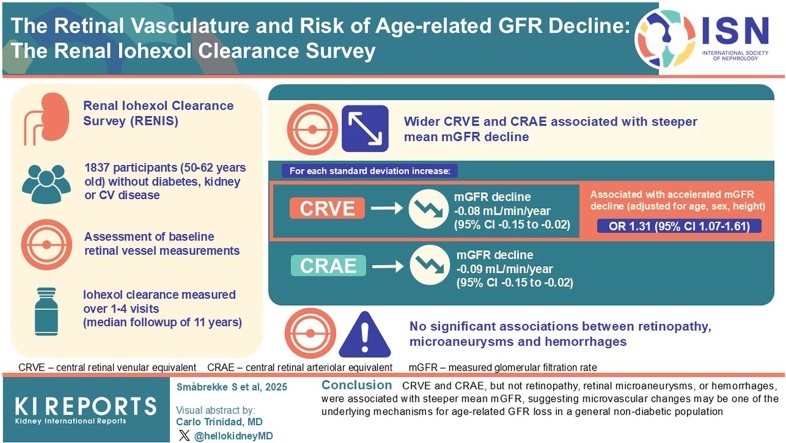

CKD is a significant public health problem affecting approximately 10% of the global population. According to the Global Burden of Disease Study, CKD is projected to become the fifth leading cause of death by 2040, primarily driven by an aging population.^1^ Advanced age is the leading risk factor for declining kidney function and the development of CKD^2^^,^^3^ in older people; nevertheless, the decline in the GFR varies among individuals. Those experiencing a more rapid decline are at significantly higher risk for CKD progression, end-stage renal disease, cardiovascular disease, and mortality.^3^, ^4^, ^5^ The disease burden and substantial healthcare costs associated with CKD underscore the need for early identification and effective management of at-risk individuals.^6^ However, the clinical management of CKD is complicated by its often asymptomatic early stages, leading to delayed diagnosis and treatment initiation.^7^

There is a strong association between kidney disease and vascular risk factors such as hyperglycemia and hypertension, both of which cause microvascular pathology. Small vessel disease primarily affects organs receiving high cardiac output, such as the kidneys and retina.^8^ In nondiabetic individuals, prediabetes and metabolic syndrome have been associated with endothelial dysfunction and inflammation—key factors thought to contribute to the development of microvascular disease in both organs.^9^ The frequent cooccurrence of diabetic nephropathy and retinopathy, often preceding significant GFR decline, suggest shared underlying mechanisms driving both renal and ocular complications in diabetes.^7^^,^^10^ However, whether similar associations between retinal vascular changes and early GFR decline exist in nondiabetic populations remains unclear.

The eyes and kidneys share several features, including similar microvascular networks, autoregulation mechanisms, and disease pathways. This is evident in the intricate vascular structures of the kidney’s glomerulus and the retinal vessels, which rely on the renin-angiotensin-aldosterone system.^11^ The transparency of the ocular media provides a noninvasive window to visualize the eye’s microvasculature, offering an early indicator of systemic microvascular disease,^9^^,^^11^^,^^12^ potentially including the renal vasculature.

Retinal microvascular changes, indicative of microvascular dysfunction, have been linked to CKD in cross-sectional studies.^12^, ^13^, ^14^ Retinal arteriolar narrowing has been associated with microalbuminuria and reduced eGFR, CKD, and end-stage renal disease.^12^^,^^13^^,^^15^^,^^16^ However, this association has not been consistently replicated in longitudinal studies.^17^, ^18^, ^19^ Similarly, evidence about the relationship between retinal venular changes and CKD remains conflicting across cross-sectional and longitudinal studies.^18^^,^^20^, ^21^, ^22^ It is possible that although retinal arteriolar narrowing and retinal venular widening and CKD share similar pathophysiological mechanisms, they may not be causally related, explaining the lack of prospective association.^18^^,^^21^^,^^23^, ^24^, ^25^ Moreover, previous studies relied on estimated GFR (eGFR) based on serum creatinine (eGFRcre) or cystatin C (eGFRcysC), which may be influenced by non-GFR–related factors, leading to imprecision and bias.^26^, ^27^, ^28^, ^29^, ^30^ Therefore, high-quality prospective studies are needed to investigate a possible link between retinal vascular changes and kidney function decline.

To the best of our knowledge, our study provides the first prospective longitudinal investigation of the associations between retinal vessel measurements and change in GFR in the general nondiabetic population. We measured the GFR (mGFR) using iohexol clearance to study the associations between retinal vessel diameters and retinopathy and age-related GFR decline over 11 years in a population-based cohort of middle-aged participants. We hypothesized that retinal vascular measurements and retinopathy are associated with age-related GFR decline, providing a potential early marker for CKD development in the general population.

## Methods

### Study Population

**Figure 1:**
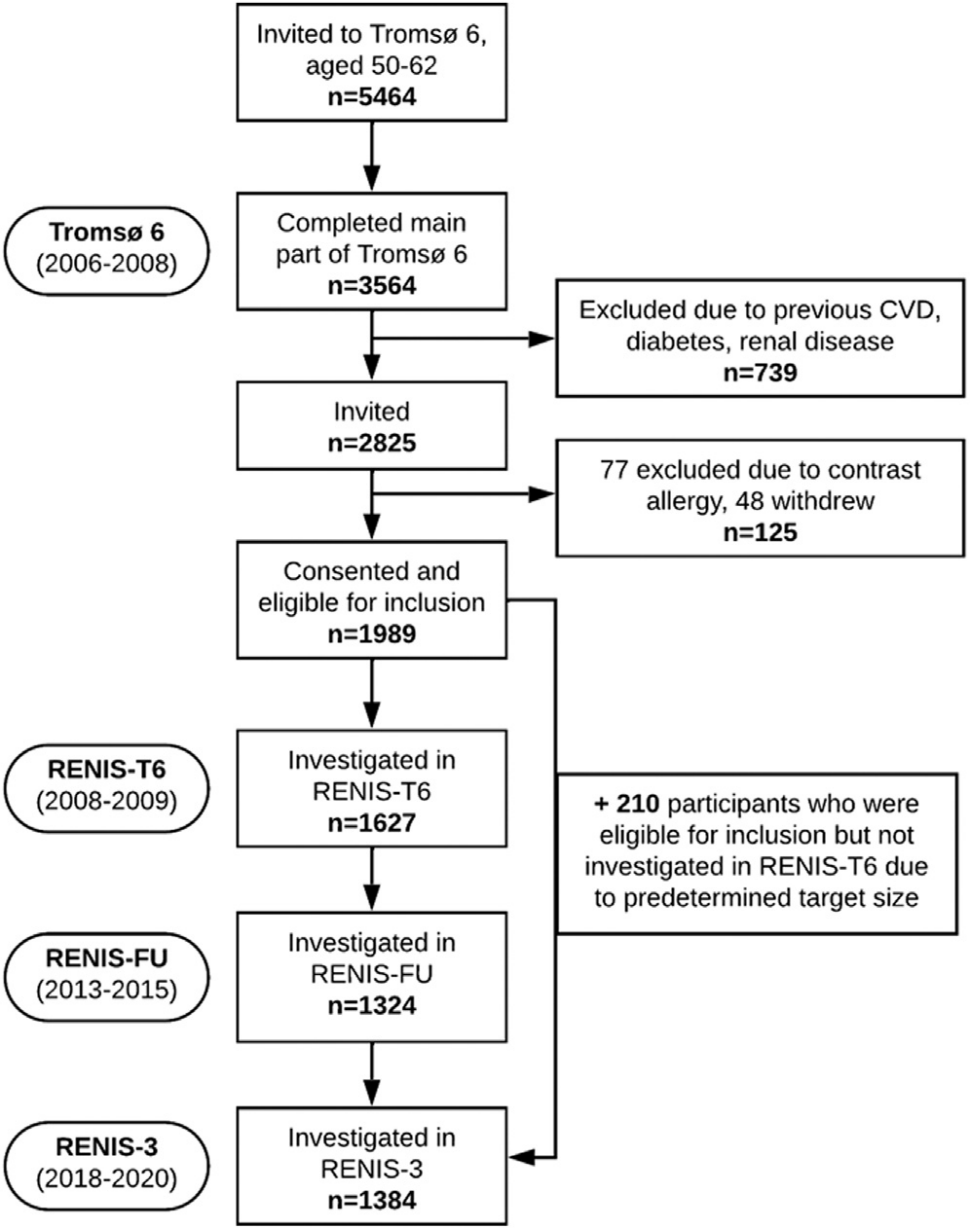
Flow chart of the study participants in the Renal Iohexol Clearance Survey from Tromsø 6 (RENIS-T6), the first follow-up (RENIS-FU) and third Renal Iohexol Clearance Survey (RENIS-3). Participants were recruited from the Tromsø 6 study.

The Tromsø Study is a longitudinal periodic health survey investigating representative samples from the general population of Tromsø, a municipality in Northern Norway. In the RENIS-T6 (2007–2009), a substudy of the sixth Tromsø study (Tromsø 6), GFR was measured in a representative sample of 1627 participants aged 50 to 62 years without self-reported diabetes, kidney disease, or cardiovascular disease, described in detail elsewhere.^31^ A total of 1324 participants (81%) and 1174 participants (72% of 1627) had a follow-up GFR measurement in the RENIS follow-up study (RENIS-FU in 2013–2015) and RENIS-3 (2018–2020), respectively (Figure 1). To minimize internal selection bias, we invited an additional 353 participants to RENIS-3 who were eligible for RENIS-T6 but not included at that time (in accordance with the study size target).^32^ Two hundred ten were included, bringing the total number of participants with GFR measurement data in RENIS-3 to 1384. Overall, 1837 participants have at least 1 GFR measurement across the RENIS studies (Supplementary Figure S1). The median duration of follow-up was 10.9 years (range: 4.4–12.8 years).

### Baseline Data and Measurements

The retinal measurements, urine samples, and questionnaires from baseline were conducted as part of the Tromsø 6 study within 1 to 2 months before the RENIS-T6. All GFR and other assessments in the RENIS study were conducted between 8:00 AM and 10:00 AM, with participants fasting at the Clinical Research Unit of the University Hospital of North Norway. Participants in RENIS-T6, RENIS-FU, and RENIS-3 completed a comprehensive questionnaire covering various topics, including physical activity, comorbidities, diet, quality of life, medications, hospital admissions, and tobacco and alcohol consumption.

### Retinal Vessel Diameters and Retinopathy

Retinal imaging was conducted using a Visucam PRONM (Carl Zeiss Meditec, Jena, Germany) retinal camera. Vessel diameters within 0.5 to 1 disc diameter from the optic disc margin were measured with computer assistance.^33^ The diameters of the 6 largest vessels of each type were aggregated to calculate the CRAE and the CRVE.^34^ Despite the absence of diabetes at baseline, retinopathy grading was performed for all participants based on "The International Clinical Diabetic Retinopathy and Diabetic Macular Edema Disease Severity Scales,"^35^ with the worst eye determining the grade. Counts of microaneurysms, hemorrhages, and soft exudates were summed up across both eyes. A dichotomous variable for retinopathy (yes/no) was created using a score > 0 on “The International Clinical Diabetic Retinopathy and Diabetic Macular Edema Disease Severity Scale.” Separate categorical variables for microaneurysms and hemorrhages were created based on their counts, categorizing participants by absence (0) or presence (1, > 1) of each. Further details have been previously published.^36^

### Measurements of GFR and Albuminuria

mGFR was assessed using a single-sample iohexol-clearance method.^31^ Five milliliter of iohexol (Omnipaque, 300 mg/ml) was injected via a Teflon catheter into an antecubital vein. Serum iohexol levels were measured at the optimal time to determine GFR using formulas by Jacobsson.^37^ The analytical coefficient of variation was 3.0% in RENIS-T6 and 3.1% in RENIS-FU,^31^ with intraindividual day-to-day GFR variation at 4.2%.^38^ External quality control for the measurements was managed by Equalis (Equalis AB, Uppsala, Sweden). To effectively compare GFR between individuals, it is crucial to consider body size. Traditionally, GFR is normalized to body surface area (BSA) and standardized to 1.73 m^2^. However, this approach has faced criticism because of its variability and may not be necessary for longitudinal studies.^39^^,^^40^ Therefore, in this study, we utilized both non-BSA–adjusted mGFR and the traditional BSA-adjusted mGFR to conduct comprehensive analyses and overcome the limitations associated with BSA-adjusted equations.

Three consecutive first-void morning urine samples were collected and immediately tested with a dipstick. On the same day, albumin and creatinine were analyzed using commercial kits for the Tromsø 6 study, RENIS-FU, and RENIS-3.^41^

### Statistical Analysis

Continuous variables were reported as mean with SD for normally distributed data and median with interquartile range for skewed data. Categorical variables were presented as percentages. Serum triglycerides were log-transformed to reduce skewness. CRAE and CRVE were transformed into standardized z-scores.

The relationships between the independent variables CRVE, CRAE, retinopathy, microaneurysm, and hemorrhages, and the annual change in the dependent variable mGFR were analyzed using separate linear mixed models with random intercepts and slopes, using an unstructured covariance matrix. The analysis included all 1837 participants, regardless of whether they had 1, 2, or 3 GFR testing visits. This approach was possible because the linear mixed regression model effectively handles missing GFR data for participants with fewer than 3 visits.^42^ The regression coefficient for the interaction between time and the independent variable represents the variable’s effect on the mGFR change rate (Table 1). A negative coefficient signifies a steeper decline with an increase in the independent variable.

**Table 1:** Associations between retinal vessel parameters and retinopathy and non-BSA adjusted mGFR change rates in linear mixed models

	Model 1		Model 2		Model 3		Model 4	
	Coef (95% CI)	*P* value	Coef (95% CI)	*P* value	Coef (95% CI)	*P* value	Coef (95% CI)	*P* value
CRAE, per SD	−0.07 (−0.13 to 0.00)	0.051	−0.09 (−0.17 to −0.02)	0.009	−0.09 (−0.16 to −0.02)	0.014	−0.09 (−0.15 to −0.02)	0.007
CRVE, per SD	−0.12 (−0.19 to −0.05)	0.001	−0.10 (−0.17 to −0.03)	0.006	−0.09 (−0.16 to −0.02)	0.009	−0.08 (−0.15 to −0.02)	0.012
Hemorrhages								
0	Ref.		Ref.		Ref.		Ref.	
1	−0.10 (−0.34 to 0.14)	0.42	−0.10 (−0.33 to 0.14)	0.42	−0.08 (−0.32 to 0.15)	0.49	−0.07 (−0.30 to 0.16)	0.56
>1	−0.02 (−0.61 to 0.56)	0.94	0.08 (−0.50 to 0.67)	0.77	0.06 (−0.52 to 0.64)	0.85	0.07 (−0.51 to 0.65)	0.81
Microaneurysms								
0	Ref.		Ref.		Ref.		Ref.	
1	0.02 (−0.33 to 0.38)	0.90	0.05 (−0.29 to 0.40)	0.76	0.07 (−0.28 to 0.42)	0.69	0.09 (−0.26 to 0.43)	0.59
>1	−0.43 (−1.30 to 0.44)	0.34	−0.28 (−1.14 to 0.58)	0.52	−0.26 (−1.11 to 0.60)	0.55	−0.16 (−1.00 to 0.69)	0.71
Retinopathy (yes/no)	−0.05 (−0.25 to 0.14)	0.61	−0.03 (−0.22 to 0.16)	0.77	−0.02 (−0.21 to 0.18)	0.86	−0.00 (−0.20 to 0.19)	0.97

CI, confidence interval; Coeff, coefficient; CRAE, central retinal arteriolar equivalent; CRP, C-reactive protein; CRVE, central retinal venular equivalent; HDL, high-density lipoprotein; LDL, low-density lipoprotein; mGFR, measured glomerular filtration rate; MMP-7, matrix metalloproteinase–7; non-BSA adjusted, non-body surface area adjusted; Ref., reference; UACR, urinary albumin-creatinine ratio.

The coefficients reflect interaction terms with observational time.

Model 1 adjusted for sex, age, and height. Model 2 adjusted for model 1 and weight, smoking, ambulatory systolic blood pressure, LDL, HDL, triglycerides, antihypertensive medication, and exercise. Model 3 adjusted for model 2 and fasting serum glucose. Model 4 adjusted for model 3 and high-sensitive CRP, MMP7, and UACR.

Directed acyclic graphs informed the selection of regression models to ensure robust adjustment for confounding and avoid bias from noncausal pathways.^43^ Our analysis included 4 nested models with variables hypothesized to be confounders, with the corresponding directed acyclic graphs for model 4 provided in Supplementary Figure S2. Model 1 was adjusted for age, sex, and height. Model 2 additionally included weight, smoking status, ambulatory systolic blood pressure, low-density lipoprotein and high-density lipoprotein cholesterol, triglycerides, and antihypertensive medication (use of angiotensin-converting enzyme inhibitors, angiotensin receptor 2 blockers, diuretics, calcium blockers, betablockers or other antihypertensive medication [yes/no]). Model 3 further adjusted for fasting glucose and physical exercise. Model 4 included model 3 and the inflammatory marker, high-sensitivity C-reactive protein; the fibrotic marker, matrix metalloproteinase–7; and urinary albumin-creatinine ratio as an indicator of endothelial dysfunction. These were included based on the hypothesis that inflammation, fibrosis, and endothelial dysfunction play a pivotal role in developing microvascular pathology.

Separate multiple logistic regression models were used to examine the associations of CRVE, CRAE, retinopathy, microaneurysms, and hemorrhages with the dependent dichotomous variable, accelerated mGFR decline. This variable was defined as the 10% of participants with the steepest annual decline rates, as determined by the linear mixed model and described in detail in a previous publication.^44^ The 1410 participants with at least 2 visits with GFR measurements were included in these analyses.

We conducted comparative analyses using eGFRcre and eGFRcysC (Supplementary Tables S3 and S4).

There was missing data for 7.9% of retinal vessel measurements and 8.4% for retinopathy grading (Supplementary Table S1). Following the current guidelines,^45^ missing data were imputed using multiple imputation techniques in STATA/MP (StataCorp LP, College Station, TX). The mixed model analyses were performed on 50 imputed datasets. The imputation model is described in a previous publication.^46^ Statistical significance was set at *P* < 0.05.

## Results

The study population consisted of 1837 persons with 4423 GFR measurements. The baseline characteristics are shown in Table 2. The mean (SD) baseline age was 58.2 (3.9) years and 51% were women. The mean (SD) BSA-adjusted mGFR at baseline was 94.0 (14.4) ml/min per 1.73 m^2^, and non-BSA adjusted GFR was 104.0 (20.1) ml/min. There were 205 (12 %) participants with retinopathy at baseline. The correlation between CRVE and CRAE was 0.62.^47^ No statistically significant association was found between CRVE or CRAE and baseline mGFR, as previously reported.^48^

**Table 2:** Baseline Characteristics of the Renal Iohexol Clearance Survey

Characteristics	All participants (*n*=1837)
Age, yr	58.2 ± 3.8
Sex, men *n* (%)	836 (47)
Weight, kg	79.2 ± 14.4
Height, cm	170.3 ± 8.8
BMI, kg/m^2^	27.2 ± 4.0
Current smoking, *n* (%)	384 (21)
BSA-adjusted mGFR, ml/min per 1.73 m^2^	94.0 ± 14.4
Non-BSA-adjusted mGFR, ml/min	104.0 ± 20.1
Urine ACR, mg/mmol (IQR)	0.23 (0.1-0.5)
CKD-EPI eGFR_cre_	94.8 ± 9.5
CKD-EPI eGFR_cysC_	105.3 ± 12.3
CKD-EPI eGFR_cysC-cre_	102.6 ± 11.5
Ambulatory BP measurements, mm Hg	
Daytime SBP	130.2 ± 13.2
Daytime DBP	82.1 ± 8.7
Nighttime SBP	111.0 ± 12.4
Nighttime DBP	66.4 ± 8.5
Antihypertensive medication, *n* (%)	299 (16)
Total cholesterol, mmol/l	5.7 ± 1.0
HDL cholesterol mmol/l	1.5 ± 0.4
LDL cholesterol, mmol/l	3.7 ± 0.9
Fasting triglycerides, mmol/l	1.2 ± 0.7
Fasting serum glucose, mmol/l	5.4 ± 0.6
CRVE, μm	212.711 ± 21.7
CRAE, μm	140.387 ± 14.8
Retinopathy, *n* (%)	205 (12)
Hemorrhages, *n* (%)	
0	1522 (91)
1	136 (8)
>1	19 (1)
Microaneurysm, *n* (%)	
0	1611 (96)
1	57 (3)
>1	9 (1)
Cotton wool spots, *n* (%)	
0	1674 (99.8)
1	2 (0.1)
>1	1 (0.1)

ACR, albumin-to-creatinine ratio; BP, blood pressure; BMI, body mass index, DBP, diastolic BP; eGFR, estimated glomerular filtration rate; HDL, high-density lipoprotein; IQR, interquartile range; LDL, low-density lipoprotein; mGFR, measured glomerular filtration rate; SBP, systolic BP.

Estimates are mean ± SD, median (interquartile range), or numbers (%). There were 210 participants with missing baseline mGFR.

The mean mGFR change rate was −1.2 ml/min/yr, calculated using a linear mixed model adjusted for baseline age, sex, height, and weight. In Table 1, we show the association between CRVE, CRAE, hemorrhages, microaneurysms, retinopathy, and GFR change rates. Wider CRVE and CRAE were associated with a steeper rate of GFR decline, estimated using linear mixed models. Specifically, each SD increase in CRVE was associated with a decrease of −0.08 ml/min/yr (95% CI:− 0.15 to −0.02, *P* = 0.012) in mGFR, and each SD increase in CRAE was associated with a decline of −0.09 ml/min/yr (95% CI:−0.15 to −0.02, *P* = 0.007) in mGFR in the fully adjusted models. There were no statistically significant interactions between sex and CRVE, CRAE, retinopathy, microaneurysm, and hemorrhages for change in mGFR rates in the mixed linear models.

For comparison, we repeated the analysis using BSA-adjusted mGFR, eGFRcre, and eGFRcysC (Supplementary Tables S2–S4, respectively). For BSA-adjusted mGFR, we found results similar to those of non-BSA-adjusted mGFR. For eGFRcre, only CRVE showed a significant association in model 1; and for eGFRcysC, significant associations were found for CRAE and CRVE across models 2 to 4, similar to the results for mGFR.

There was no significant association between retinopathy, retinal microaneurysms, or retinal hemorrhages and changes in mGFR. However, using eGFRcre and eGFRcysC, retinal hemorrhages were significantly associated with eGFR decline in models 1 to 4 in the group with the count of 1 hemorrhage, detailed in Supplementary Table S3.

In Table 3, we show multiple logistic regression analyses with odds ratios for accelerated GFR decline. The threshold to define accelerated GFR decline, using the 10th percentile of the GFR slope distribution, was −1.87 ml/min/yr (*n* = 140). Wider CRVE was associated with an increased risk of accelerated GFR decline in model 1 adjusted for sex, age, and height, with odds ratio = 1.31 (95% CI: 1.07–1.61, *P* = 0.008), but not in the models with additional adjustment for weight, smoking, ambulatory systolic blood pressure, low-density lipoprotein, high-density lipoprotein, triglycerides, use of antihypertensive medication, exercise, fasting serum glucose, high-sensitivity C-reactive protein, matrix metalloproteinase–7, and urinary albumin-creatinine ratio. CRAE was not associated with an increased risk for accelerated GFR decline. Neither was retinopathy, retinal hemorrhages, or retinal microaneurysm.

**Table 3:** Multiple logistic regression analyses with ORs for accelerated non-BSA adjusted mGFR decline

	Model 1		Model 2		Model 3		Model 4	
	OR (95% CI)	*P* value	OR (95% CI)	*P* value	OR (95% CI)	*P* value	OR (95% CI)	*P* value
CRAE, per SD	0.99 (0.81–1.22)	0.95	1.20 (0.94–1.52)	0.14	1.11 (0.87–1.42)	0.41	1.14 (0.88–1.47)	0.33
CRVE, per SD	1.31 (1.07–1.61)	0.008	1.24 (0.98–1.56)	0.07	1.19 (0.94–1.52)	0.15	1.19 (0.93–1.53)	0.17
Hemorrhages								
0	Ref.		Ref.		Ref.		Ref.	
1	1.20 (0.64–2.28)	0.57	1.25 (0.62–2.50)	0.53	1.14 (0.55–2.40)	0.72	1.11 (0.51–2.40)	0.80
>1	1.71 (0.48–6.09)	0.41	0.71 (0.18–2.83)	0.63	0.84 (0.12–3.52)	0.81	0.88 (0.21–3.71)	0.86
Microaneurysms								
0	Ref.		Ref.		Ref.		Ref.	
1	0.64 (0.22–1.90)	0.42	0.60 (0.19–1.88)	0.38	0.62 (0.20–1.94)	0.41	0.60 (0.19–1.92)	0.39
>1	1.82 (0.32–10.33)	0.50	1.10 (0.19–6.40)	0.92	0.67 (0.07–6.9)	0.74	0.70 (0.07–7.06)	0.76
Retinopathy (yes/no)	1.19 (0.70–2.02)	0.52	1.07 (0.61–1.90)	0.81	1.05 (0.58–1.92)	0.86	1.07 (0.61–1.90)	0.81

CI, confidence interval; CRAE, central retinal arteriolar equivalent; CRP, C-reactive protein; CRVE, central retinal venular equivalent; HDL, high-density lipoprotein; LDL, low-density lipoprotein; mGFR, measured glomerular filtration rate; MMP-7, matrix metalloproteinase–7; non-BSA-adjusted, non-body surface area adjusted; OR, odds ratio; Ref., reference.

Model 1 was adjusted for sex, age, and height. Model 2 was adjusted for model 1, including weight, smoking, ambulatory systolic blood pressure, LDL, HDL, triglycerides, antihypertensive meditation, and exercise. Model 3 was adjusted for model 2 and fasting serum glucose. Model 4 was adjusted for model 3 and high-sensitive CRP, MMP7, and UACR (urinary albumin-creatinine ratio).

## Discussion

In a general population devoid of self-reported kidney disease, diabetes, or cardiovascular disease, we observed significant associations between wider CRVE and CRAE and a more rapid decline in mGFR. A wider CRVE, but not CRAE, was also associated with the dichotomous outcome accelerated decline in mGFR in a model adjusted only for sex, age, and height. Retinopathy, retinal microaneurysms, and retinal hemorrhages showed no association with the mean declines or accelerated decline rates of mGFR.

Previous studies have examined the relationship between retinal vessel diameters, retinopathy, and incident CKD or eGFR decline, with inconsistent findings.^17^^,^^18^^,^^21^^,^^23^^,^^24^^,^^49^, ^50^, ^51^, ^52^ Furthermore, to our knowledge, no previous studies specifically focused on nondiabetic populations, underscoring a significant gap in the existing literature.

Wider CRVE is hypothesized to reflect endothelial dysfunction and inflammation,^53^ contributing to basement membrane thickening and vascular leakage, features often seen in CKD.^7^ Our finding of wider CRVE contrasts with other longitudinal studies. For instance, one study excluding participants with baseline CKD initially found an association between wider CRVE and incident CKD over a 6-year follow-up period. However, when stratified by diabetes status, this association did not persist in nondiabetic participants.^21^ This study relied on eGFR measurements, limiting comparability to our cohort and mGFR-based results; and the shorter follow-up period might mean that CRVE-related changes had not fully developed in their cohort, potentially explaining the differing findings to our study. Two other studies that included participants with diabetes reported no associations between wider CRVE and incident CKD.^18^^,^^23^ Both these studies were based on single eGFR measurements at each visit, potentially reducing precision and leading to misclassification of CKD status because factors such as hydration state, medication use, and dietary protein intake can transiently increase eGFR values. In addition, one of the studies adjusted for fellow retinal vessel caliber (e.g., CRAE in models including CRVE and vice versa),^23^ which is questionable because of the moderately strong correlation between the variables as found in our study.

The finding of wider CRAE associated with GFR decline was unexpected, because previous longitudinal studies have linked narrower CRAE to incident CKD, lower eGFR, or eGFR decline.^21^^,^^23^^,^^49^, ^50^, ^51^ Narrower CRAE is thought to reflect dysregulation of the renin-angiotensin-aldosterone system and elevated endothelin, a potent vasoconstrictor.^11^ Mechanisms for wider CRAE are less clear, though a study found a U-shaped relationship between retinal arteriolar caliber and albuminuria.^15^ Elevated endothelin, associated with CKD progression,^7^^,^^53^ may also contribute, although its impact could be limited by our cohort's low prevalence of CKD.

Diabetes, a primary factor in CKD and early microvascular damage,^54^ complicates assessments in the general population because findings may not generalize to nondiabetic groups. Previous studies investigating retinal vessel diameters and incident CKD or reduced eGFR included diabetic participants at baseline,^21^^,^^23^^,^^49^^,^^51^ which may impact results because of diabetes's essential effects on retinal microvascular health and kidney function. We excluded persons with preexisting diabetes, deliberately avoiding the impact of diabetes’ direct implications of retinal vascular health on renal outcomes in the general population.

In contrast to our findings, where retinopathy was not associated with mGFR decline, other studies have reported associations between retinopathy, retinal hemorrhages, and microaneurysms with incident CKD and eGFR decline.^17^^,^^21^^,^^52^ However, these studies included diabetic participants at baseline, again limiting comparability to our findings. In our relatively healthy nondiabetic cohort, only a few participants had retinopathy, with most cases limited to a single microaneurysm or hemorrhage (Table 2). This may reduce the statistical power to detect associations, as reflected in the relatively wide CIs.

Retinal microvascular abnormalities, along with ocular conditions such as glaucoma and cataracts, are influenced by factors such as aging, diabetes, hypertension, smoking, hyperlipidemia, and systemic inflammation. However, we found that CRVE and CRAE were associated with GFR decline even after adjusting for these risk factors, including 24-hour ambulatory systolic blood pressure. Endothelial dysfunction and systemic inflammation, linked with retinal vascular changes, are crucial in the development and progression of CKD.^12^^,^^55^^,^^56^ However, our findings remained unchanged after adjusting for low-grade inflammation, a fibrotic marker; and urinary albumin-creatinine ratio, which serves as a marker of endothelial dysfunction, in model 4.

Previous studies have relied on eGFR, typically derived from creatinine and/or cystatin C, despite these markers' well-known biases and imprecision, because they are influenced by factors other than GFR. Therefore, the differences between mGFR and eGFR are not random errors but reflect variables that may be associated with disease.^48^ Our eGFR results differed from those obtained with mGFR. This suggests that eGFRcre and eGFRcysC do not reliably substitute for mGFR when studying the longitudinal association between retinal vessel measurement, retinopathy, and kidney function; and may, in part, explain the conflicting evidence reported in longitudinal studies.^17^^,^^18^^,^^21^^,^^23^^,^^24^^,^^49^, ^50^, ^51^, ^52^

A significant strength of our study is the repeated GFR measurements within a well-documented, healthy cohort without preexisting diabetes, kidney, or cardiovascular disease. A limitation of this study is the inclusion of Caucasians only, which limits generalizability to other ethnic groups. The study's observational design means that causality inferences cannot be made. Although dropout rates were low, selection bias cannot be entirely excluded if less healthy individuals were more likely to exit the study. The impact of competing risks, such as mortality, was minimal, with only 5% of participants dying during the study period. Although we used directed acyclic graphs to minimize confounding, residual confounding may still exist because of unknown mechanisms.

## Conclusion

In the middle-aged nondiabetic general population, increasing CRVE and CRAE are associated with a steeper mGFR decline; however, retinopathy, retinal microaneurysms, and hemorrhages are not. These findings suggest that common systemic microvascular processes likely contribute to the development of microvascular damage in both the eyes and kidneys.

## Disclosure

All the authors declared no competing interests.

## Supplementary Material

Supplementary File (PDF) **Supplementary References. Figure S1.** The total study population of the Renal Iohexol Clearance Survey. **Figure S2.** Directed acyclic graph (DAG) model 4. **Table S1.** Baseline characteristics of the Renal Iohexol Clearance Survey, including missing data. **Table S2.** Associations between retinal vessel parameters and retinopathy and BSA-adjusted mGFR (ml/min per 1.73 m^2^) changes in linear mixed models. **Table S3.** Associations between retinal vessel parameters and retinopathy and eGFRcre (CKD-EPI equation) change rates in linear mixed models. **Table S4.** Associations between retinal vessel parameters and retinopathy and eGFRcysC (CKD-EPI equation) change rates in linear mixed models. Strobe Checklist.

## Footnotes

Supplementary File (PDF)

**Supplementary References.**
**Figure S1.** The total study population of the Renal Iohexol Clearance Survey. **Figure S2.** Directed acyclic graph (DAG) model 4. **Table S1.** Baseline characteristics of the Renal Iohexol Clearance Survey, including missing data. **Table S2.** Associations between retinal vessel parameters and retinopathy and BSA-adjusted mGFR (ml/min per 1.73 m^2^) changes in linear mixed models. **Table S3.** Associations between retinal vessel parameters and retinopathy and eGFRcre (CKD-EPI equation) change rates in linear mixed models. **Table S4.** Associations between retinal vessel parameters and retinopathy and eGFRcysC (CKD-EPI equation) change rates in linear mixed models. Strobe Checklist.

## References

[1] Lye W.K., Paterson E., Patterson C.C. (2021). A systematic review and participant-level meta-analysis found little association of retinal microvascular caliber with reduced kidney function. Kidney Int.

[2] Agarwal R., Bunaye Z., Bekele D.M., Light R.P. (2008). Competing risk factor analysis of end-stage renal disease and mortality in chronic kidney disease. Am J Nephrol.

[3] Eriksen B.O., Palsson R., Ebert N. (2020). GFR in healthy aging: An individual participant data meta-analysis of iohexol clearance in European population-based cohorts. J Am Soc Nephrol.

[4] Matsushita K., van der Velde M., Astor B.C. (2010). Association of estimated glomerular filtration rate and albuminuria with all-cause and cardiovascular mortality in general population cohorts: A collaborative meta-analysis. Lancet.

[5] Hommos M.S., Glassock R.J., Rule A.D. (2017). Structural and functional changes in human kidneys with healthy aging. J Am Soc Nephrol.

[6] Weiner D.E., Tabatabai S., Tighiouart H. (2006). Cardiovascular outcomes and all-cause mortality: Exploring the interaction between CKD and cardiovascular disease. Am J Kidney Dis.

[7] Aronov M., Allon R., Stave D. (2021). Retinal vascular signs as screening and prognostic factors for chronic kidney disease: A systematic review and meta-analysis of current evidence. J Pers Med.

[8] Abbas K., Lu Y., Bavishi S. (2022). A simple review of small vessel disease manifestation in the brain, retina, and kidneys. J Clin Med.

[9] Wong T.Y., McIntosh R. (2005). Systemic associations of retinal microvascular signs: A review of recent population-based studies. Ophthalmic Physiol Opt.

[10] Cankurtaran V., Inanc M., Tekin K., Turgut F. (2020). Retinal microcirculation in predicting diabetic nephropathy in Type 2 diabetic patients without retinopathy. Ophthalmologica.

[11] Farrah T.E., Dhillon B., Keane P.A., Webb D.J., Dhaun N. (2020). The eye, the kidney, and cardiovascular disease: Old concepts, better tools, and new horizons. Kidney Int.

[12] Sabanayagam C., Shankar A., Koh D. (2009). Retinal microvascular caliber and chronic kidney disease in an Asian population. Am J Epidemiol.

[13] Sabanayagam C., Tai E.S., Shankar A., Lee J., Sun C., Wong T.Y. (2009). Retinal arteriolar narrowing increases the likelihood of chronic kidney disease in hypertension. J Hypertens.

[14] Liew G., Mitchell P., Wong T.Y., Wang J.J. (2012). Retinal microvascular signs are associated with chronic kidney disease in persons with and without diabetes. Kidney Blood Press Res.

[15] Awua-Larbi S., Wong T.Y., Cotch M.F. (2011). Retinal arteriolar caliber and urine albumin excretion: The Multi-Ethnic Study of Atherosclerosis. Nephrol Dial Transplant.

[16] Baumann M., Burkhardt K., Heemann U. (2014). Microcirculatory marker for the prediction of renal end points: A prospective cohort study in patients with chronic kidney disease stage 2 to 4. Hypertension.

[17] Edwards M.S., Wilson D.B., Craven T.E. (2005). Associations between retinal microvascular abnormalities and declining renal function in the elderly population: The cardiovascular health study. Am J Kidney Dis.

[18] Sabanayagam C., Shankar A., Klein B.E. (2011). Bidirectional association of retinal vessel diameters and estimated GFR decline: The Beaver Dam CKD Study. Am J Kidney Dis.

[19] Grunwald J.E., Pistilli M., Ying G.S. (2014). Retinopathy and progression of CKD: The CRIC study. Clin J Am Soc Nephrol.

[20] Bao S., Huang W., Liang Y. (2015). Retinal vessel diameter and chronic kidney disease in rural china: A cross-sectional study. Medicine (Baltimore).

[21] Yip W., Ong P.G., Teo B.W. (2017). Retinal vascular imaging markers and incident chronic kidney disease: A prospective cohort study. Sci Rep.

[22] Grunwald J.E., Pistilli M., Ying G.S. (2021). Progression of retinopathy and incidence of cardiovascular disease: Findings from the chronic renal insufficiency Cohort Study. Br J Ophthalmol.

[23] Yau J.W., Xie J., Kawasaki R. (2011). Retinal arteriolar narrowing and subsequent development of CKD Stage 3: The Multi-Ethnic Study of Atherosclerosis (MESA). Am J Kidney Dis.

[24] Grunwald J.E., Pistilli M., Ying G.S. (2019). Association between progression of retinopathy and concurrent progression of kidney disease: Findings from the chronic renal insufficiency cohort (CRIC) study. JAMA Ophthalmol.

[25] Yip W., Sabanayagam C., Teo B.W. (2015). Retinal microvascular abnormalities and risk of renal failure in Asian populations. PLoS One.

[26] Peralta C.A., Jacobs D.R., Katz R. (2012). Association of pulse pressure, arterial elasticity, and endothelial function with kidney function decline among adults with estimated GFR >60 mL/min/1.73 m(2): The Multi-Ethnic Study of Atherosclerosis (MESA). Am J Kidney Dis.

[27] Rule A.D., Bailey K.R., Lieske J.C., Peyser P.A., Turner S.T. (2013). Estimating the glomerular filtration rate from serum creatinine is better than from cystatin C for evaluating risk factors associated with chronic kidney disease. Kidney Int.

[28] Mathisen U.D., Melsom T., Ingebretsen O.C. (2011). Estimated GFR associates with cardiovascular risk factors independently of measured GFR. J Am Soc Nephrol.

[29] Knight E.L., Verhave J.C., Spiegelman D. (2004). Factors influencing serum cystatin C levels other than renal function and the impact on renal function measurement. Kidney Int.

[30] Stevens L.A., Schmid C.H., Greene T. (2009). Factors other than glomerular filtration rate affect serum cystatin C levels. Kidney Int.

[31] Eriksen B.O., Stefansson V.T.N., Jenssen T.G. (2016). Elevated blood pressure is not associated with accelerated glomerular filtration rate decline in the general non-diabetic middle-aged population. Kidney Int.

[32] Melsom T., Norvik J.V., Enoksen I.T. (2022). Sex differences in age-related loss of kidney function. J Am Soc Nephrol.

[33] von Hanno T., Bertelsen G., Sjolie A.K., Mathiesen E.B. (2014). Retinal vascular calibres are significantly associated with cardiovascular risk factors: The Tromso Eye Study. Acta Ophthalmol.

[34] Knudtson M.D., Lee K.E., Hubbard L.D., Wong T.Y., Klein R., Klein B.E.K. (2003). Revised formulas for summarizing retinal vessel diameters. Curr Eye Res.

[35] Wilkinson C.P., Ferris F.L., Klein R.E. (2003). Proposed international clinical diabetic retinopathy and diabetic macular edema disease severity scales. Ophthalmology.

[36] Bertelsen G., Erke M.G., von Hanno T. (2013). The Tromso Eye Study: Study design, methodology and results on visual acuity and refractive errors. Acta Ophthalmol.

[37] Jacobsson L. (1983). A method for the calculation of renal clearance based on a single plasma sample. Clin Physiol.

[38] Eriksen B.O., Stefansson V.T.N., Jenssen T.G. (2017). High ambulatory arterial stiffness index is an independent risk factor for rapid age-related glomerular filtration rate decline in the general middle-aged population. Hypertension.

[39] Eriksen B.O., Melsom T., Mathisen U.D., Jenssen T.G., Solbu M.D., Toft I. (2011). GFR normalized to total body water allows comparisons across genders and body sizes. J Am Soc Nephrol.

[40] Delanaye P., Krzesinski J.M. (2011). Indexing of renal function parameters by body surface area: Intelligence or folly?. Nephron Clin Pract.

[41] Solbu M.D., Kronborg J., Jenssen T.G. (2009). Albuminuria, metabolic syndrome and the risk of mortality and cardiovascular events. Atherosclerosis.

[42] Twisk J., de Boer M., de Vente W., Heymans M. (2013). Multiple imputation of missing values was not necessary before performing a longitudinal mixed-model analysis. J Clin Epidemiol.

[43] Tennant P.W.G., Murray E.J., Arnold K.F. (2021). Use of directed acyclic graphs (DAGs) to identify confounders in applied health research: Review and recommendations. Int J Epidemiol.

[44] Norvik J.V., Harskamp L.R., Nair V. (2021). Urinary excretion of epidermal growth factor and rapid loss of kidney function. Nephrol Dial Transplant.

[45] Sterne J.A., White I.R., Carlin J.B. (2009). Multiple imputation for missing data in epidemiological and clinical research: Potential and pitfalls. BMJ.

[46] Eriksen B.O., Smabrekke S., Jenssen T.G. (2018). Office and ambulatory heart rate as predictors of age-related kidney function decline. Hypertension.

[47] Mukaka M.M. (2012). Statistics corner: A guide to appropriate use of correlation coefficient in medical research. Malawi Med J.

[48] Eriksen B.O., Lochen M.L., Arntzen K.A. (2015). Estimated and measured GFR associate differently with retinal vasculopathy in the general population. Nephron.

[49] Lim L.S., Cheung C.Y., Sabanayagam C. (2013). Structural changes in the retinal microvasculature and renal function. Invest Ophthalmol Vis Sci.

[50] Baumann M., Schwarz S., Kotliar K. (2009). Non-diabetic chronic kidney disease influences retinal microvasculature. Kidney Blood Press Res.

[51] Gu Y.M., Petit T., Wei F.F. (2016). Renal glomerular dysfunction in relation to retinal arteriolar narrowing and high pulse pressure in seniors. Hypertens Res.

[52] Hwang H.S., Kim S.Y., Hong Y.A. (2016). Clinical impact of coexisting retinopathy and vascular calcification on chronic kidney disease progression and cardiovascular events. Nutr Metab Cardiovasc Dis.

[53] Wong C.W., Wong T.Y., Cheng C.Y., Sabanayagam C. (2014). Kidney and eye diseases: Common risk factors, etiological mechanisms, and pathways. Kidney Int.

[54] Kawasaki R., Cheung N., Wang J.J. (2009). Retinal vessel diameters and risk of hypertension: The Multiethnic Study of Atherosclerosis. J Hypertens.

[55] Enoksen I.T., Svistounov D., Norvik J.V. (2022). Serum matrix metalloproteinase 7 and accelerated glomerular filtration rate decline in a general non-diabetic population. Nephrol Dial Transplant.

[56] Schei J., Stefansson V.T.N., Eriksen B.O. (2017). Association of TNF Receptor 2 and CRP with GFR decline in the general nondiabetic population. Clin J Am Soc Nephrol.

