# Supplementary material for: The Retinal Vasculature and Risk of Age-Related GFR Decline — The Renal Iohexol Clearance Survey
Source: Kidney Int Rep. 2025 Feb 14;10(5):1384–92. doi: 10.1016/j.ekir.2025.02.006 (PMC12142612; doi:10.1016/j.ekir.2025.02.006)
Supplement: Supplementary File (PDF) — Supplementary References. Figure S1. The total study population of the Renal Iohexol Clearance Survey. Figure S2. Directed acyclic graph (DAG) model 4. Table S1. Baseline characteristics of the Renal Iohexol Clearance Survey, including missing data. Table S2. Associations between retinal vessel parameters and retinopathy and BSA-adjusted mGFR (ml/min per 1.73 m2) changes in linear mixed models. Table S3. Associations between retinal vessel parameters and retinopathy and eGFRcre (CKD-EPI equation) change rates in linear mixed models. Table S4. Associations between retinal vessel parameters and retinopathy and eGFRcysC (CKD-EPI equation) change rates in linear mixed models. Strobe Checklist. [file mmc1.pdf]

# Supplementary material

## The Retinal Vasculature and Risk of Age-related GFR Deline – The Renal Iohexol Clearance Survey

Silje Småbrekke[1,2,5], Karl Marius Brobakk[1,2,4], Nikoline Rinde[1,2], Therese Von Hanno[2,6], Geir Bertelsen[3,7], Bjørn Odvar Eriksen[1,2,4], Toralf Melsom[1,2,4]

[1]Metabolic and Renal Research Group, UiT The Arctic University of Norway, Hansine Hansens veg 18, N-9019 Tromsø, Norway

[2]Department of Clinical Medicine, UiT The Arctic University of Norway, Hansine Hansens veg 18 N-9019 Tromsø, Norway

[3]Department of Community Medicine, UiT The Arctic University of Norway, Hansine Hansens veg 18 N-9019 Tromsø, Norway

[4]Section of Nephrology, University Hospital of North Norway, Sykehusvegen 38, N-9019 Tromsø, Norway

[5]Section of Radiology, University Hospital of North Norway, Sykehusvegen 38, N-9019, Tromsø, Norway

[6]Department of Ophthalmology, Nordland Hospital Trust, Parkveien 95, N-8005, Bodø, Norway

[7]Section of Ophthalmology, University Hospital of North Norway, Sykehusvegen 38, N-9019 Tromsø, Norway

## Contents

**Figure S1:** The total study population in the Renal Iohexol Clearance Survey (RENIS) with at least one GFR measurement ........ 3

**Figure S2:** Directed acyclic graph (DAG) model 4. ........ 4

**Table S1:** Baseline characteristics of the Renal Iohexol Clearance Survey, including missing ........ 5

**Table S2:** Associations between retinal vessel parameters and retinopathy and BSA-adjusted mGFR (ml/min/1.73m$^2$) changes rates in linear mixed models ........ 6

**Table S3:** Associations between retinal vessel parameters and retinopathy and eGFRcre (CKD-EPI equation) changes rates in linear mixed models ........ 7

**Table S4:** Associations between retinal vessel parameters and retinopathy and eGFRcysC (CKD-EPI equation) changes rates in linear mixed models ........ 8

**STROBE Statement—checklist of items that should be included in reports of observational studies** ........ 9

**Supplementary references** ........ 11

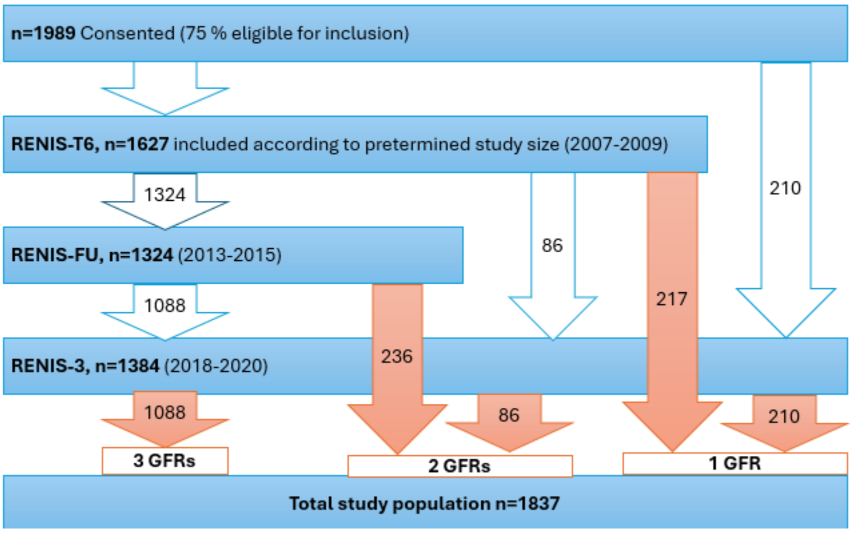


**Figure S1:** The total study population in the Renal Iohexol Clearance Survey (RENIS) with at least one GFR measurement

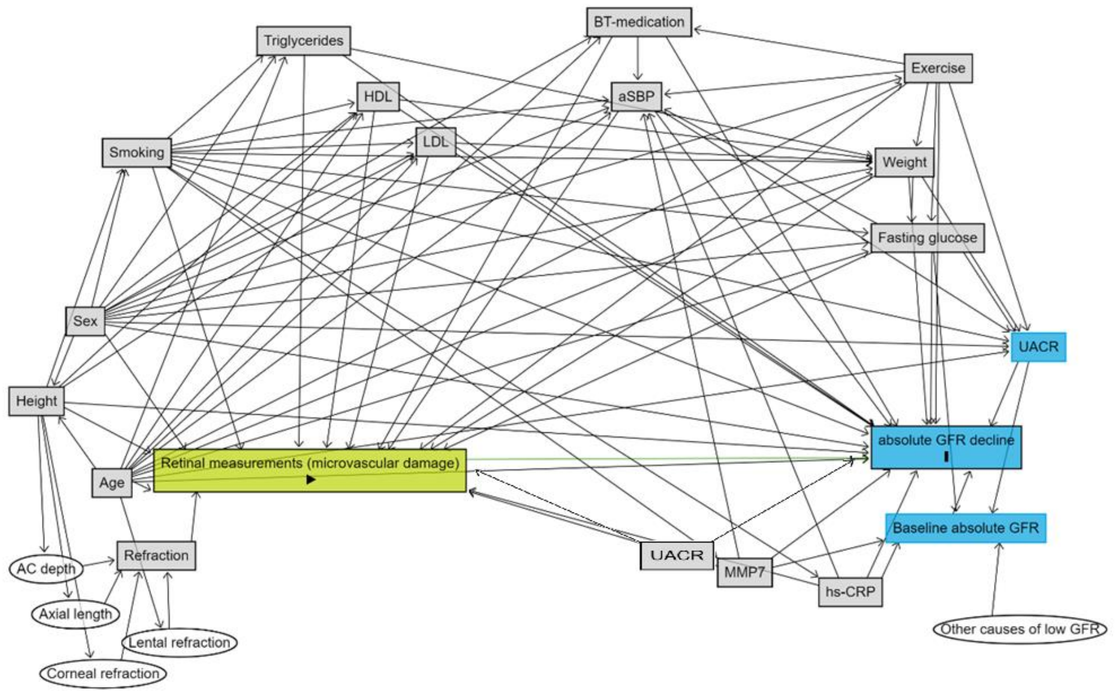


**Figure S2:** Directed acyclic graph (DAG) model 4.

DAGs (Directed Acyclic Graphs) visually model the assumptions about the structure and relationships between independent variables (such as retinal vessel measurements, hemorrhages, microaneurysms, and retinopathy) and the dependent variable (decline in non-BSA-adjusted mGFR). This visual representation was created using daggity.net, and it helps clarify theoretical pathways, highlighting potential confounders and mediators and thereby providing a clearer understanding of the underlying assumptions in our analysis (S1, S2). Within the DAGs: Exposure is indicated by green boxes. Outcomes are shown as blue boxes with a dark border. Ancestors of the outcome are depicted in blue boxes without a dark border. Adjusted variables are represented in grey boxes. Unobserved variables appear as elliptical white shapes. Causal pathways are marked with green lines.

In model 4, we ended up adjusting for age, sex, height, weight, smoking, ambulatory SBP, BT-medication, triglycerides, LDL, HDL, fasting glucose, exercise, MMP7, hs-CRP and UACR, as all these variables are confounding the relationship between retinal measurements and non-BSA-adjusted mGFR decline. We did not adjust for  baseline non-BSA-adjusted mGFR, nor could we adjust for the unobserved valuables AC depth, axial length, corneal refraction, lental refraction, or other causes of low GFR.

**Table S1:** Baseline characteristics of the Renal Iohexol Clearance Survey, including missing

| Characteristics | All participants (*n*=1837) | Missing |
|---|---|---|
| Age, yr | 58.2 ± 3.8 | 0 |
| Sex, men *n* (%) | 836 (47) | 0 |
| Weight, kg | 79.2 ± 14.4 | 0 |
| Height, cm | 170.3 ± 8.8 | 0 |
| BMI, kg/m$^2$ | 27.2 ± 4.0 | 0 |
| Current smoking, *n* (%) | 384 (21) | 5 |
| BSA-adjusted mGFR, ml/min/1.73m$^2$ | 94.0 ± 14.4 | 210 |
| Non-BSA-adjusted mGFR, ml/min | 104.0 ± 20.1 | 210 |
| Urine ACR, mg/mmol (IQR) | 0.23 (0.1-0.5) | 6 |
| CKD-EPI $eGFR_{cre}$ | 94.8 ± 9.5 | 210 |
| CKD-EPI $eGFR_{cysC}$ | 105.3 ± 12.3 | 0 |
| CKD-EPI $eGFR_{cysC\text{-}crea}$ | 102.6 ± 11.5 | 0 |
| Ambulatory BP measurements, mm Hg | | |
| Daytime SBP | 130.2 ± 13.2 | 229 |
| Daytime DBP | 82.1 ± 8.7 | 229 |
| Nighttime SBP | 111.0 ± 12.4 | 229 |
| Nighttime DBP | 66.4 ± 8.5 | 229 |
| Antihypertensive medication, *n* (%) | 299 (16) | 5 |
| Total cholesterol, mmol/l | 5.7 ± 1.0 | 0 |
| HDL cholesterol mmol/l | 1.5 ± 0.4 | 0 |
| LDL cholesterol, mmol/l | 3.7 ± 0.9 | 0 |
| Fasting triglycerides, mmol/l | 1.2 ± 0.7 | 214 |
| Fasting serum glucose, mmol/l | 5.4 ± 0.6 | 210 |
| CRVE, µm | 212.711 ± 21.7 | 151 |
| CRAE, µm | 140.387 ± 14.8 | 151 |
| Retinopathy, n (%) | 205 (12) | 160 |
| Hemorrhages, n (%) | | 160 |
| 0 | 1522 (91) | |
| 1 | 136 (8) | |
| >1 | 19 (1) | |
| Microaneurysm, n (%) | | 160 |
| 0 | 1611 (96) | |
| 1 | 57 (3) | |
| >1 | 9 (1) | |
| Cotton wool spots, n (%) | | 160 |
| 0 | 1674 (99.8) | |
| 1 | 2 (0.1) | |
| >1 | 1 (0.1) | |

**Table S2:** Associations between retinal vessel parameters and retinopathy and BSA-adjusted mGFR (ml/min/1.73m$^2$) changes rates in linear mixed models

| | Model 1 | | Model 2 | | Model 3 | | Model 4 | |
|---|---|---|---|---|---|---|---|---|
| | Coef (95% CI) | P-value | Coef (95% CI) | P-value | Coef (95% CI) | P-value | Coef (95% CI) | P-value |
| CRVE* | -0.08 (-0.14 to -0.02) | 0.014 | -0.08 (-0.15 to -0.02) | 0.011 | -0.08 (-0.14 to -0.01) | 0.017 | -0.09 (-0.15 to -0.2) | 0.008 |
| CRVE* | -0.11 (-0.17 to -0.05) | 0.000 | -0.09 (-0.15 to -0.02) | 0.008 | -0.08 (-0.15 to -0.02) | 0.012 | -0.08 (-0.15 to -0.02) | 0.012 |
| Hemorrhages | | | | | | | | |
| 0 | Ref. | | Ref. | | Ref. | | Ref. | |
| 1 | -0.08 (-0.30-0.13) | 0.45 | -0.08 (-0.30-0.13) | 0.459 | -0.07 (-0.28-0.15) | 0.54 | -0.08 (-0.29-0.14) | 0.48 |
| >1 | -0.03 (-0.57-0.51) | 0.91 | 0.08 (-0.45-0.61) | 0.764 | 0.06 (-0.47-0.59) | 0.83 | 0.09 (-0.44-0.62) | 0.74 |
| Microaneurysms | | | | | | | | |
| 0 | Ref. | | Ref. | | Ref. | | Ref. | |
| 1 | 0.00 (-0.32-0.33) | 0.97 | 0.03 (-0.29-0.35) | 0.86 | 0.04 (-0.28-0.36) | 0.80 | 0.05 (-0.27-0.37) | 0.75 |
| >1 | -0.32 (-1.12-0.48) | 0.44 | -0.19 (-0.98-0.59) | 0.63 | -0.17 (-0.96-0.61) | 0.67 | -0.10 (-0.88-0.68) | 0.79 |
| Retinopathy | -0.05 (-0.23-0.13) | 0.60 | -0.09 (-0.21-0.15) | 0.75 | -0.02 (-0.20-0.16) | 0.84 | -0.02 (-0.20-0.16) | 0.83 |

Each row represents separate linear mixed models. *CRVE and CRAE were transformed into standardized Z-scores (one standard deviation increase or decrease from the mean). The coefficients reflect interaction terms with observational time. A positive coefficient reflects an increase in the independent variable is associated with an increase in GFR, and a negative coefficient reflects that as the independent variable increases, GFR decreases

Model 1 adjusted for sex and age.
Model 2 adjusted for model 1+ BMI, smoke, ambulatory systolic blood pressure, LDL (low-density lipoprotein), HDL (high-density lipoprotein), triglycerides, use of antihypertensive medication (angiotensin-converting enzyme inhibitors, angiotensin receptor II blockers, diuretics, calcium blockers, beta-blockers, or other antihypertensive medications (yes/no)) and exercise.
Model 3 adjusted for the same variables as model 2 + fasting glucose.
Model 4 adjusted for model 3 + hs-CRP (high-sensitivity C-reactive protein), MMP7 (matrix metalloproteinase 7), and UACR (urinary albumin-creatinine ratio).

Abbreviations: Coeff, coefficient; CI, confidence interval, CRVE, central retinal venular equivalent; CRAE, central retinal arteriolar equivalent

**Table S3:** Associations between retinal vessel parameters and retinopathy and eGFRcre (CKD-EPI equation) changes rates in linear mixed models

| | Model 1 | | Model 2 | | Model 3 | | Model 4 | |
|---|---|---|---|---|---|---|---|---|
| | Coef (95% CI) | P-value | Coef (95% CI) | P-value | Coef (95% CI) | P-value | Coef (95% CI) | P-value |
| CRVE* | -0.04 (-0.08-0.00) | 0.06 | -0.04 (-0.09-0.00) | 0.07 | -0.04 (-0.09-0.00) | 0.08 | -0.04 (-0.09-0.00) | 0.07 |
| CRVE* | -0.05 (-0.09 to -0.01) | 0.02 | -0.04 (-0.08-0.01) | 0.09 | -0.04 (-0.08-0.01) | 0.11 | -0.03 (-0.08-0.02) | 0.19 |
| Hemorrhages | | | | | | | | |
| 0 | Ref. | | Ref. | | Ref. | | Ref. | |
| 1 | -0.22 (-0.37 to -0.06) | 0.005 | -0.22 (-0.37 to -0.06) | 0.006 | -0.21 (-0.3 to -0.06) | 0.006 | -0.21 (-0.36 to -0.05) | 0.009 |
| >1 | -0.08 (-0.46-0.30) | 0.68 | -0.02 (-0.40-0.36) | 0.92 | -0.02 (-0.40-0.36) | 0.92 | 0.00 (-0.38-0.38) | 0.99 |
| Microaneurysms | | | | | | | | |
| 0 | Ref. | | Ref. | | Ref. | | Ref. | |
| 1 | 0.17 (-0.06-0.40) | 0.15 | 0.19 (-0.04-0.41) | 0.11 | -0.19 (-0.04-0.42) | 0.10 | 0.23 (-0.01-0.46) | 0.06 |
| >1 | -0.52 (-1.08-0.04) | 0.07 | -0.45 (-1.01-0.11) | 0.11 | -0.44 (-0.99-0.12) | 0.12 | 0.23 (-0.01-0.46) | 0.06 |
| Retinopathy | -0.11 (-0.24-0.02) | 0.09 | -0.10 (-0.23-0.02) | 0.12 | -0.10 (-0.22-0.03) | 0.13 | -0.08 (-0.21-0.04) | 0.20 |

Each row represents separate linear mixed models. *CRVE and CRAE were transformed into standardized Z-scores (one standard deviation increase or decrease from the mean). The coefficients reflect interaction terms with observational time. A positive coefficient reflects an increase in the independent variable, which is associated with an increase in GFR, and a negative coefficient reflects that as the independent variable increases, GFR decreases.

Model 1 adjusted for sex and age.
Model 2 adjusted for model 1+ BMI, smoke, ambulatory systolic blood pressure, LDL (low-density lipoprotein), HDL (high-density lipoprotein), triglycerides, use of antihypertensive medication (angiotensin-converting enzyme inhibitors, angiotensin receptor II blockers, diuretics, calcium blockers, beta-blockers, or other antihypertensive medications (yes/no)) and exercise.
Model 3 adjusted for the same variables as model 2 + fasting glucose.
Model 4 was adjusted for model 3 + hs-CRP (high-sensitivity C-reactive protein), MMP7 (matrix metalloproteinase 7), and UACR (urinary albumin-creatinine ratio).

Abbreviations: eGFRcre, estimated glomerular filtration rate based on creatinine; CKD-EPI, Chronic Kidney Disease Epidemiology Collaboration; Coeff, coefficient; CI, confidence interval, CRVE, central retinal venular equivalent; CRAE, central retinal arteriolar equivalent

**Table S4:** Associations between retinal vessel parameters and retinopathy and eGFRcysC (CKD-EPI equation) changes rates in linear mixed models

| | Model 1<br>Coef (95% CI) | P-value | Model 2<br>Coef (95% CI) | P-value | Model 3<br>Coef (95% CI) | P-value | Model 4<br>Coef (95% CI) | P-value |
|---|---|---|---|---|---|---|---|---|
| CRAE* | -0.04 (-0.09-0.01) | 0.15 | -0.06 (-0.12 to -0.01) | 0.02 | -0.06 (-0.12 to -0.01) | 0.02 | -0.06 (-0.12 to -0.01) | 0.03 |
| CRVE* | -0.05 (-0.11-0.00) | 0.05 | -0.06 (-0.12 to -0.01) | 0.02 | -0.07 (-0.12 to -0.01) | 0.02 | -0.06 (-012 to -0.01) | 0.02 |
| Hemorrhages | | | | | | | | |
| 0 | Ref. | | Ref. | | Ref. | | Ref. | |
| 1 | -0.22 (-0.40 to -0.03) | 0.02 | -0.21 (-0.40 to -0.03) | 0.03 | -0.22 (-0.40 to -0.03) | 0.03 | -0.22 (-0.40 to -0.03) | 0.03 |
| >1 | 0.09 (-0.37-0.54) | 0.71 | 0.12 (-0.34-0.58) | 0.62 | 0.12 (-0.34-0.58) | 0.62 | 0.14 (-0.32-0.60) | 0.56 |
| Microaneurysms | | | | | | | | |
| 0 | Ref. | | Ref. | | Ref. | | Ref. | |
| 1 | 0.07 (-0.21-0.34) | 0.63 | 0.08 (-0.20-0.36) | 0.55 | 0.08 (-0.20-0.36) | 0.55 | 0.09 (-0.18-0.37) | 0.51 |
| >1 | -0.01 (-0.70-0.68) | 0.98 | 0.04 (-0.65-0.73) | 0.92 | 0.04 (-0.65-0.73) | 0.91 | 0.07 (-0.62-0.76) | 0.84 |
| Retinopathy | -0.10 (-0.25-0.06) | 0.22 | -0.09 (-0.24-0.06) | 0.25 | -0.09 (-0.24-0.06) | 0.25 | -0.09 (-0.24-0.07) | 0.28 |

Each row represents separate linear mixed models. *CRVE and CRAE were transformed into standardized Z-scores (one standard deviation increase or decrease from the mean). The coefficients reflect interaction terms with observational time. A positive coefficient reflects an increase in the independent variable, which is associated with an increase in GFR, and a negative coefficient reflects that as the independent variable increases, GFR decreases.

Model 1 adjusted for sex and age.
Model 2 adjusted for model 1+ BMI, smoke, ambulatory systolic blood pressure, LDL (low-density lipoprotein), HDL (high-density lipoprotein), triglycerides, use of antihypertensive medication (angiotensin-converting enzyme inhibitors, angiotensin receptor II blockers, diuretics, calcium blockers, beta-blockers, or other antihypertensive medications (yes/no)) and exercise.
Model 3 adjusted for the same variables as model 2 + fasting glucose.
Model 4 was adjusted for model 3 + hs-CRP (high-sensitivity C-reactive protein), MMP7 (matrix metalloproteinase 7), and UACR (urinary albumin-creatinine ratio).

Abbreviations: eGFRcysC, estimated glomerular filtration rate based on cystatin C; CKD-EPI, Chronic Kidney Disease Epidemiology Collaboration; Coeff, coefficient; CI, confidence interval; CRVE, central retinal venular equivalent; CRAE, central retinal arteriolar equivalent

**STROBE Statement—checklist of items that should be included in reports of observational studies**

| | Item No | Recommendation | Page no |
|---|---|---|---|
| **Title and abstract** | 1 | (*a*) Indicate the study's design with a commonly used term in the title or the abstract | 2 |
| | | (*b*) Provide in the abstract an informative and balanced summary of what was done and what was found | 2 |
| **Introduction** | | | |
| Background/rationale | 2 | Explain the scientific background and rationale for the investigation being reported | 3-4 |
| Objectives | 3 | State specific objectives, including any prespecified hypotheses | 3-4 |
| **Methods** | | | |
| Study design | 4 | Present key elements of study design early in the paper | 4 |
| Setting | 5 | Describe the setting, locations, and relevant dates, including periods of recruitment, exposure, follow-up, and data collection | 4-6 |
| Participants | 6 | (*a*) *Cohort study*—Give the eligibility criteria, and the sources and methods of selection of participants. Describe methods of follow-up | 4 |
| | | (*b*) *Cohort study*—For matched studies, give matching criteria and number of exposed and unexposed | |
| Variables | 7 | Clearly define all outcomes, exposures, predictors, potential confounders, and effect modifiers. Give diagnostic criteria, if applicable | 6-7 |
| Data sources/ measurement | 8* | For each variable of interest, give sources of data and details of methods of assessment (measurement). Describe comparability of assessment methods if there is more than one group | 5-6 |
| Bias | 9 | Describe any efforts to address potential sources of bias | 7 |
| Study size | 10 | Explain how the study size was arrived at | 4 |
| Quantitative variables | 11 | Explain how quantitative variables were handled in the analyses. If applicable, describe which groupings were chosen and why | 5-6 |
| Statistical methods | 12 | (*a*) Describe all statistical methods, including those used to control for confounding | 6-7 |
| | | (*b*) Describe any methods used to examine subgroups and interactions | 7 |

| | | | |
|---|---|---|---|
| | | (*c*) Explain how missing data were addressed | 7 |
| | | (*d*) *Cohort study*—If applicable, explain how loss to follow-up was addressed | |
| | | (*e*) Describe any sensitivity analyses | |
| **Results** | | | |
| Participants | 13 | (a) Report numbers of individuals at each stage of study—eg numbers potentially eligible, examined for eligibility, confirmed eligible, included in the study, completing follow-up, and analysed | 4-5 |
| | | (b) Give reasons for non-participation at each stage | 5 |
| | | (c) Consider use of a flow diagram | Figure 1 |
| Descriptive data | 14 | (a) Give characteristics of study participants (eg demographic, clinical, social) and information on exposures and potential confounders | Table 1 |
| | | (b) Indicate number of participants with missing data for each variable of interest | Table S1 |
| | | (c) *Cohort study*—Summarise follow-up time (eg, average and total amount) | 4-5, Figure S1 |
| Outcome data | 15 | *Cohort study*—Report numbers of outcome events or summary measures over time | 5-6 |
| Main results | 16 | (*a*) Give unadjusted estimates and, if applicable, confounder-adjusted estimates and their precision (eg, 95% confidence interval). Make clear which confounders were adjusted for and why they were included | 6-8 |
| | | (*b*) Report category boundaries when continuous variables were categorized | 5 |
| | | (*c*) If relevant, consider translating estimates of relative risk into absolute risk for a meaningful time period | |
| Other analyses | 17 | Report other analyses done—eg analyses of subgroups and interactions, and sensitivity analyses | 7 |
| **Discussion** | | | |
| Key results | 18 | Summarise key results with reference to study objectives | 8-9 |
| Limitations | 19 | Discuss limitations of the study, taking into account sources of potential bias or imprecision. Discuss both direction and magnitude of any potential bias | 11 |
| Interpretation | 20 | Give a cautious overall interpretation of results considering objectives, limitations, multiplicity of analyses, results from similar studies, and other relevant evidence | 8-11 |
| Generalisability | 21 | Discuss the generalisability (external validity) of the study results | 11 |
| **Other information** | | | |

| | | | |
|---|---|---|---|
| Funding | 22 | Give the source of funding and the role of the funders for the present study and, if applicable, for the original study on which the present article is based | |

**Note:** An Explanation and Elaboration article discusses each checklist item and gives methodological background and published examples of transparent reporting. The STROBE checklist is best used in conjunction with this article (freely available on the Web sites of PLoS Medicine at http://www.plosmedicine.org/, Annals of Internal Medicine at http://www.annals.org/, and Epidemiology at http://www.epidem.com/). Information on the STROBE Initiative is available at www.strobe-statement.org.

## Supplementary References


S1. Suttorp MM, Siegerink B, Jager KJ, Zoccali C, Dekker FW. Graphical presentation of confounding in directed acyclic graphs. Nephrology, dialysis, transplantation : official publication of the European Dialysis and Transplant Association - European Renal Association. 2015;30(9):1418-23.

S2. Textor J, van der Zander B, Gilthorpe MS, Liskiewicz M, Ellison GT. Robust causal inference using directed acyclic graphs: the R package 'dagitty'. Int J Epidemiol. 2016;45(6):1887-94.
